# Leisure time exercise and depressive symptoms in sedentary workers: exploring the effects of exercise volume and social context

**DOI:** 10.3389/fpsyt.2025.1570681

**Published:** 2025-04-10

**Authors:** Jianjiang Zhang, Jiyang Yang, Siping Chen, Chan Feng, Shaoying Wang

**Affiliations:** ^1^ Medical College, Ningde Normal University, Ningde, China; ^2^ School of General Education, Xinjiang Career Technical College, Kuitun, China; ^3^ Medical College, Shaoguan University, Shaoguan, China

**Keywords:** leisure-time exercise, depressive symptoms, sedentary workers, group exercise, individual exercise

## Abstract

**Background:**

Workers in sedentary occupations often engage in prolonged periods of low physical activity, which may be associated with depressive symptoms. Leisure-time exercise plays a significant role in alleviating these symptoms. Previous studies have shown that adults who engage in physical exercise report fewer depressive symptoms than those who do not. However, the relationship between exercise volume and mental health remains inconsistent. Leisure-time exercise can be categorized into individual and group exercise. Despite its potential importance, little is known about the differential effects of individual and group exercise on depressive symptoms in sedentary occupational populations. The objective of this study was to analyze the association between leisure-time exercise volume and depressive symptoms in sedentary workers, as well as to evaluate the disparities in the effects of individual and group exercise on depressive symptoms.

**Methods:**

From September to October 2024, a cross-sectional survey was conducted to collect data from sedentary workers. The participants’ sociodemographic characteristics, exercise patterns, exercise volume, and depressive symptoms were gathered. Chi-square tests and hierarchical logistic regression were employed to analyze the obtained data.

**Results:**

Of the 1,277 respondents, 13.16% reported depressive symptoms. The prevalence of depressive symptoms was higher in those with low exercise volume than in those with medium or high exercise volume. Medium and high exercise volumes were associated with a lower risk of depressive symptoms, with odds ratios (OR) of 0.517 and 0.559, respectively. Group exercisers reported fewer depressive symptoms than individual exercisers, with an OR of 0.624.

**Conclusion:**

The benefits of leisure-time exercise on depressive symptoms in sedentary workers do not always increase with higher exercise volume. Additionally, sedentary workers who participated in group exercise exhibited a lower risk of depressive symptoms than those who participated in individual exercise.

## 1 Introduction

As society evolves and technology advances, sedentary behavior has become a defining characteristic of many professions (1). For instance, telephone customer service representatives, information technology professionals, office staff, and other occupational groups now commonly engage in prolonged sitting during work, which has gradually become a normative aspect of their daily tasks. This type of work is characterized by long periods of sitting and low physical activity. A large cross-sectional analysis of 4,436 employees showed that office workers reported sitting for 376 ± 106 minutes per workday, which accounts for 60% of total sitting time per day (2). The pattern of sedentary behavior has raised widespread concern regarding its potential health risks (3). Sedentary behavior is typically defined as low energy expenditure, with energy expenditure levels equal to or less than 1.5 times the basal metabolic rate (4). The health risks associated with sedentary behavior include cardiovascular disease (5), metabolic disorders (6), musculoskeletal issues (7), and mental health concerns (8). Recent evidence suggests an association between sedentary behavior and depressive symptoms (9). Occupation-related sedentary behavior reduces physical activity time, which has been shown to positively affect mental health by regulating neurotransmitter levels and endocrine function (10). Sedentary behavior may also be linked to loneliness, work burnout, and social isolation, potentially exacerbating mental health issues (11). Furthermore, sedentary behavior is frequently accompanied by high levels of stress and cognitive load at work (12). The effects of these factors, whether individually or in combination, may exacerbate the negative impact on depressive symptoms in occupational populations.

Depressive symptoms represent a prevalent mental disorder, affecting approximately 322 million individuals globally (13). The Global Burden of Disease Study reported that depressive symptoms constitute the leading cause of mental health-related disability burden globally (14). Occupational factors represent significant risk factors for depressive symptoms (15). Depressive symptoms not only diminish the quality of life of working individuals, but may also contribute to increased absenteeism (16) and reduced job satisfaction (17). The indirect economic losses caused by depressive symptoms in the working population cannot be overlooked, affecting both enterprises and society (18).

To mitigate the adverse effects of occupational sedentary behavior on mental health, leisure-time exercise represents an important intervention strategy. Studies indicate that regular physical activity alleviates depressive symptoms through various mechanisms, including enhancing endorphin secretion, regulating neurotransmitters (19), and improving self-efficacy (20). Leisure-time exercise is crucial for maintaining and enhancing the mental health, Bélair showed that there was a significant association between reduced leisure-time physical activity and increased depressive symptoms, especially in adolescents (21). Huang pointed out that reduced high-intensity exercise and increased sedentary behavior would aggravate depressive symptoms based on the US National Health and Nutrition Examination Surveys (NHANES 2017–2020) data (22). Conclusions regarding the relationship between exercise volume and mental health remains inconsistent. One study found a positive correlation between leisure-time exercise volume and mental health, suggesting that increased exercise volume leads to greater mental health benefits (23). However, a cross-sectional study examining the relationship between physical exercise and mental health in over 1.2 million individuals in the United States found that exercising for more than 6 hours per week is linked to poorer mental health (24). Exercise can be categorized into individual and group exercises. Individual exercise includes activities performed alone, such as running, cycling, and swimming, and is characterized by low social engagement. It emphasizes self-control and autonomy, fosters psychological satisfaction, enhances personal control and self-confidence, and may be particularly suitable for participants who prefer solitude but lack the social support typically provided by group activities. Group exercise includes activities performed with others, such as football, basketball, and volleyball, and is associated with higher social interaction. Group exercise typically requires the formation of social networks, which can foster social interaction, enhance collective belonging and support, and provide participants with greater emotional support (25). This may be beneficial in reducing loneliness and social avoidance, which are related to depressive symptoms.

While prior studies have established a link between physical activity and mental well-being, gaps remain in understanding the appropriate exercise volume needed to “compensate” the adverse psychological effects of prolonged sedentary behavior. Additionally, the differential impact of individual and group exercise on mental health in occupational settings has been largely unexplored. Given the relationship between exercise and mental health, analyzing the relationship between leisure-time exercise volume and depressive symptoms in sedentary workers, as well as comparing the effects of individual and group exercise on depressive symptoms, may help to inform intervention strategies and guide clinical practices. This study aims to analyze the relationship between leisure-time exercise volume and depressive symptoms in sedentary workers and compare the effects of individual and group exercise on depressive symptoms during leisure time, to provide a scientific basis for mental health intervention strategies. To address the aforementioned research objectives, this study proposes the following hypotheses:

- The risk of depressive symptoms in sedentary workers decreases as leisure-time exercise volume increases.
- Compared to individual exercise, sedentary workers engaging in group exercise experience a lower risk of depressive symptoms.

## 2 Methods and materials

### 2.1 Participants

This study adopts the classification method proposed by Gao et al. (26), where the term “sedentary workers” refers to individuals who engage in prolonged sitting at work and exhibit low levels of physical activity. Bank employees serve as a typical representation of the sedentary workers. They typically remain seated at their desks for the majority of their working hours, utilizing computers for tasks such as word processing, data analysis, and customer service. During work, they often have limited opportunities to walk or stretch. This study employs a cross-sectional design with cluster sampling. Participants in this study were recruited from a large state-owned bank in Fujian Province in China. The sample size for simple random sampling was calculated using the following formula:


$$\text{n}=\frac{z_{\frac{\text{a}}{2}}{\;}^{2}\times p\times (1-p)}{d^{2}}$$

In the formula, *n* represents the sample size, with a 95% confidence level, $z_{\frac{\text{a}}{2}}^{2}$ =1.96, *p* is the overall positive rate, based on the preliminary survey (*p* = 0.10), and the allowable error (*d* = 0.05), *n* was 139. The design effect for cluster sampling was calculated using the following formula: DEFF = 1 + (*m* - 1) × ICC. Here, DEFF refers to the design effect, *m* represents the average sample size per cluster, and ICC refers to the intraclass correlation coefficient. According to a preliminary survey, *m* = 65 and ICC = 0.10, yielding a DEFF value of 7.40. Thus, the required sample size for cluster sampling is 7.40 × 139 = 1,029 cases. Considering a 20% invalid response rate, the final sample size requirement is approximately 1,235 cases. (1) Inclusion criteria: (i) male or female; (ii) employed in the job for over one year; (iii) aged between 18 and 60 years; (iv) capable of completing the questionnaire independently. (2) Exclusion criteria: (i) a history of organic brain disorders, schizophrenia, or bipolar disorder; (ii) individuals with severe heart, liver, kidney, lung, or other major organ diseases, or malignant tumors that hinder regular physical activity; (iii) pregnant or lactating women; (iv) individuals not at work due to illness or other reasons during the survey; (v) individuals with suicidal tendencies. Before the survey, all participants were informed of the study’s purpose and the instructions for completing the questionnaire. Additionally, participants were assured that the confidentiality of their data would be maintained. Data were collected from participants between September and October 2024 using the online survey platform “www.wjx.cn” (a Chinese-based online questionnaire platform). This study was reviewed and approved by the Ethics Committee of Ningde Normal University (NDNU-LL-202106). All participants understood the nature and potential impact of the study, provided informed consent, and completed the questionnaire independently.

### 2.2 Sociodemographic characteristics

The questionnaire gathered sociodemographic characteristics from the respondents, including gender (male, female), age (≤30 years, 31-40 years, 41-50 years, ≥51 years), marital status (unmarried, married, divorced, widowed), education level (junior high school or below, high school, and college or above), and average monthly income (≤5,000 RMB, 5,000-10,000 RMB, ≥10,001 RMB). Additionally, the questionnaire assessed health-related behavioral habits, including smoking status (current smoker, former smoker, non-smoker) and drinking status (current drinker, former drinker, non-drinker). Furthermore, the questionnaire collected respondents’ height and weight and calculated the body mass index (BMI). BMI is calculated as follows:


$$(\text{BMI})=\frac{\text{Weight (kg)}}{{\text{ [Height (m)]}}^{2}}$$

In this formula, <18.5 is classified as underweight, 18.5–23.9 as normal weight, 24.0–27.9 as overweight, and ≥28 as obese (27).

### 2.3 Depressive symptoms

Depressive symptoms were assessed using the Patient Health Questionnaire-9 (PHQ-9), a widely utilized tool with demonstrated reliability and validity (28). The PHQ-9 consists of 9 items, and respondents select the corresponding frequency based on their experiences over the past two weeks. The scoring system is as follows: “Not at all” = 0 points, “Several days” = 1 point, “More than half the days” = 2 points, “Nearly every day” = 3 points, with a total score range of 0–27 points. A score of ≥10 points on the PHQ-9 was used as the cutoff for depressive symptom screening. The Cronbach’s α coefficient for the PHQ-9 in this study was 0.898.

### 2.4 Leisure-time exercise volume

The respondents’ leisure time exercise volume was assessed using the Chinese version Physical Activity Rating Scale-3 (PARS-3) (29). The questionnaire evaluates physical activity based on three dimensions: activity time, frequency, and intensity. Activity time is categorized into the following intervals: less than 10 minutes, 11-20 minutes, 21-30 minutes, 31-59 minutes, and more than 60 minutes per session. Frequency is classified as once a month, 2-3 times a month, 1-2 times a week, 3-5 times a week, and daily. Intensity is classified as mild (e.g., walking, radio gymnastics), low (e.g., Tai Chi), sustained moderate (e.g., cycling, running), non-persistent high (e.g., badminton, basketball), and sustained high intensity (e.g., swimming, running). The formula for calculating exercise volume is as follows:


Exercise volume=intensity × (time − 1) × frequency

Each item is scored on a range from 1 to 5 points, and the total score ranges from 0 to 100 points. Based on the score classification, low exercise volume is defined as less than 19 points, medium exercise volume as 20 to 42 points, and high exercise volume as more than 43 points. The Cronbach’s α coefficient for the PARS-3 in this study was 0.837.

### 2.5 Leisure-time exercise context

In this study, “exercise” refers to planned, organized, repetitive, and purposeful physical activity (30). Based on the classification of exercise contexts by Doré et al. (31), participants were initially asked whether they engaged in any form of leisure-time exercise (including individual or group exercise) during a typical 7-day work cycle (Monday to Sunday). Participants who did not engage in any form of exercise were not required to continue answering. If the answer is “yes,” participants were asked to select the option that best described their exercise form: exclusively individual exercise (performed alone), exclusively group exercise, or both individual and group exercise. From the perspective of whether it has social attributes, this study divided exercise categories into group exercise and individual exercise, group exercise was defined as exercise performed with at least one other person (e.g., football, basketball, volleyball), while individual exercise refer to exercise performed alone (e.g., walking, running, cycling). Considering the social context, participants who “exclusively participate in group exercise” and those who “participate in both individual and group exercise” were classified as group exercisers in this study.

### 2.6 Data analyses

Statistical analysis was performed using SPSS 26.0 (IBM Corp., Armonk, NY, USA). Cronbach’s α coefficient was computed for the PHQ-9 and PARS-3 scales. Categorical data, including gender, marital status, education level, and smoking status, were described as percentages. The chi-square test was used to compare the count data of different groups. When *P* < 0.05, further pairwise comparisons were performed, to control for type I errors, the Bonferroni correction was applied. When analyzing the factors influencing depressive symptoms, participants were classified into those with or without depressive symptoms based on a PHQ-9 score of ≥10 points. A stratified binary logistic regression model was used to calculate the OR and 95% confidence intervals (CI) for each variable. To account for the influence of covariates, this study established three models. The first model included gender, age, education level, average monthly income, smoking status, drinking status, and BMI as independent variables. The second model was based on Model 1 and incorporated varying levels of exercise volume. The third model was based on Model 2 and incorporated different exercise modalities. All statistical tests were two-tailed, except for the Bonferroni correction for *post hoc* multiple comparisons, in which α was set to 0.05.

## 3 Results

### 3.1 Sociodemographic characteristics

A total of 1,455 questionnaires were collected, with 178 questionnaires that did not meet the inclusion criteria excluded, leaving 1,277 valid responses and an effective response rate of 87.77%. Among the respondents, the highest proportions were women (53.95%), individuals in the 31-40 age group (32.81%), married individuals (75.88%), and those with a college degree or higher (55.44%). Additionally, 44.95% had an average monthly income between 5,001 and 10,000 RMB, 67.82% were non-smokers, 75.96% were non-drinkers, and 56.54% had a BMI between 18.5-23.9 (see **Table 1**). Of the 1,277 respondents, 850 (66.56%) engaged in individual exercise, while 427 (33.44%) participated in group exercise. In general, among group exercisers, individuals with higher exercise volumes, males, younger participants, and those without overweight or obesity were more likely to engage in group exercise, with significant differences observed (*P* < 0.05), as shown in **Table 1**.

**Table 1:** Comparison of sociodemographic characteristics of individual and group exercisers (N = 1277).

Variable	Classification	Total (*n*/%)	Individual exercise (*n*/%)	Group exercise (*n*/%)	χ^2^	*P* ^a^
Exercise volume	low	351 (27.49)	253 (72.08)	98 (27.92)	7.174	**0.028**
	medium	597 (46.75)	390 (65.33)	207 (34.67)		
	high	329 (25.76)	207 (62.92)	122 (37.08)		
Gender	male	588 (46.05)	371 (63.10)	217 (36.90)	5.886	**0.015**
	female	689 (53.95)	479 (69.52)	210 (30.48)		
Age (years)	≤30	330 (25.84)	195 (59.09)	135 (40.91)	26.728	**<0.001**
	31-40	419 (32.81)	262 (62.53)	157 (37.47)		
	41-50	323 (25.29)	236 (73.07)	87 (26.93)		
	≥51	205 (16.05)	157 (76.59)	48 (23.41)		
Marital status	unmarried	179 (14.02)	111 (62.01)	68 (37.99)	6.502	0.090
	married	969 (75.88)	647 (66.77)	322 (33.23)		
	divorcee	83 (6.50)	64 (77.11)	19 (22.89)		
	widowed	46 (3.60)	28 (60.87)	18 (39.13)		
Education level	junior high school or below	109 (8.54)	73 (66.97)	36 (33.03)	0.155	0.925
	high school	460 (36.02)	303 (65.87)	157 (34.13)		
	college or higher	708 (55.44)	474 (66.95)	234 (33.05)		
Average monthly income (RMB)	≤5000	472 (36.96)	321 (68.01)	151 (31.99)	0.792	0.673
	5001-10000	574 (44.95)	379 (66.03)	195 (33.97)		
	≥10001	231 (18.09)	150 (64.94)	81 (35.06)		
Smoking status	non-smoker	866 (67.82)	564 (65.13)	302 (34.87)	4.514	0.105
	former smoker	105 (8.22)	79 (75.24)	26 (24.76)		
	current smoker	306 (23.96)	207 (67.65)	99 (32.35)		
Drinking status	non-drinker	970 (75.96)	630 (64.95)	340 (35.05)		
	former drinker	175 (13.70)	123 (70.29)	52 (29.71)	5.067	0.079
	current drinker	132 (10.34)	97 (73.48)	35 (26.52)		
BMI	<18.5	74 (5.79)	46 (62.16)	28 (37.84)	8.362	**0.039**
	18.5-23.9	722 (56.54)	482 (66.76)	240 (33.24)		
	24.0-27.9	332 (26.00)	209 (62.95)	123 (37.05)		
	≥28	149 (11.67)	113 (75.84)	36 (24.16)		

^a^Bold values indicate *P*<0.05.

### 3.2 Prevalence of depressive symptoms

Among the 1,277 respondents, 168 (13.16%) had a PHQ-9 score of ≥10. Based on exercise modality, the detection rate of depressive symptoms was lower in group exercisers compared to individual exercisers, and this difference was statistically significant (χ^2^ = 8.057, *P* = 0.005), as shown in **Table 2**. Regarding exercise volume, the difference in the detection rate of depressive symptoms across different exercise volume was statistically significant (*P* < 0.05). Further pairwise comparisons revealed that the detection rate of depressive symptoms was higher in the low exercise volume group than in the medium exercise volume group (χ^2^ = 13.809, *P* < 0.001) and the high exercise volume group (χ^2^ = 9.318, *P* = 0.002). However, no statistically significant difference was found between the medium and high exercise volume groups (χ^2^ = 0.011, *P* = 0.917), as shown in **Table 2**.

**Table 2:** Comparison of the detection rate of depressive symptoms by exercise modality and volume (N = 1277).

Depressive symptoms	Total (*n*/%)	Individual exercise (*n*/%)	Group exercise (*n*/%)	Low exercise volume (*n*/%)	Medium exercise volume (*n*/%)	High exercise volume (*n*/%)
No	1109 (86.84)	722 (84.94)	387 (90.63)	283 (80.63)	533 (89.28)	293 (89.06)
Yes	168 (13.16)	128 (15.06)	40 (9.37)	68 (19.37)	64 (10.72)	36 (10.94)
χ^2^	–	8.057		16.386		
*P* ^a^	–	**0.005**		**<0.001**		

^a^Bold values indicate *P*<0.05.

### 3.3 Influencing factors of depressive symptoms

The presence or absence of depressive symptoms was set as the dependent variable (0=No, 1=Yes), and a hierarchical logistic regression was used to analyze the factors influencing depressive symptoms. Three models were developed. In Model 1, the variables that influenced depressive symptoms included gender, education level, and smoking status (*P* < 0.05). However, age, marital status, average monthly income, drinking status, and BMI did not have a significant effect (*P* > 0.05). In Model 2, in addition to the variables above, exercise volume was also found to influence depressive symptoms (*P* < 0.05). The results of the final regression model (Model 3) indicated that individuals with moderate or high exercise volume had a lower risk of depressive symptoms (*P* < 0.05). After controlling for covariates, the OR and 95% confidence intervals (CI) for individuals with medium and high exercise volume were 0.517 (0.351, 0.761) and 0.559 (0.354, 0.880), respectively. Compared to individual exercisers, group exercisers had a lower risk of depressive symptoms, with an OR (95% CI) of 0.624 (0.420, 0.927), as shown in **Table 3**.

**Table 3:** Logistic regression analysis of variable influencing depressive symptoms (N = 1277).

Model	Independent variable	*B*	OR	95% CI	*P* ^d^
Model 1 ^a^	Female (refer to male)	0.831	2.296	(1.465, 3.596)	**<0.001**
	High school (refer to junior high school or below)	-0.643	0.526	(0.303, 0.913)	**0.022**
	college or higher (refer to junior high school or below)	-0.767	0.465	(0.275, 0.786)	**0.004**
	Smoker (refer to non-smoker)	0.988	2.685	(1.770, 4.072)	**<0.001**
Model 2 ^b^	Female (refer to male)	0.811	2.251	(1.434, 3.535)	**<0.001**
	High school (refer to junior high school or below)	-0.721	0.486	(0.279, 0.848)	**0.011**
	college or higher (refer to junior high school or below)	-0.816	0.442	(0.260, 0.750)	**0.002**
	Smoker (refer to non-smoker)	0.994	2.701	(1.771, 4.119)	**<0.001**
	Medium exercise volume (refer to low exercise volume)	-0.690	0.502	(0.341, 0.738)	**<0.001**
	High exercise volume (refer to low exercise volume)	-0.619	0.538	(0.342, 0.847)	**0.007**
Model 3 ^c^	Female (refer to male)	0.760	2.138	(1.355, 3.373)	**0.001**
	High school (refer to junior high school or below)	-0.702	0.496	(0.283, 0.867)	**0.014**
	college or higher (refer to junior high school or below)	-0.806	0.447	(0.262, 0.760)	**0.003**
	Smoker (refer to non-smoker)	0.993	2.700	(1.769, 4.121)	**<0.001**
	Medium exercise volume (refer to low exercise volume)	-0.660	0.517	(0.351, 0.761)	**0.001**
	High exercise volume (refer to low exercise volume)	-0.582	0.559	(0.354, 0.880)	**0.012**

a Model 1 uses the presence or absence of depressive symptoms as the dependent variable, and gender, age, marital status, education level, smoking, alcohol consumption, and body mass index as the independent variables.

b Model 2 builds upon Model 1 by adding exercise volume as an independent variable.

c Model 3 extends Model 2 by incorporating exercise modality as an additional independent. ^d^ Bold values indicate *P*<0.05.

## 4 Discussion

Sedentary behavior in modern occupational environments poses significant public health concerns (32), with depressive symptoms in sedentary workers receiving considerable attention (33). Given the lack of physical activity in sedentary workers, engaging in exercise during leisure time represents an economical and effective intervention for mitigating depressive symptoms in these groups. Previous research has established the psychological health benefits of exercise for the general population (34). A randomized controlled trial confirmed the antidepressant effects of resistance training in young adults (35) and a prospective follow-up of 33,908 “healthy” adults for 11 years showed that regular leisure-time exercise was associated with a reduced incidence of future depression (36). However, a small amount of research has investigated whether sedentary workers require a greater volume of exercise to “compensate” for the adverse effects of sedentary behavior on mental health, or whether individual and group exercise have differential impacts on depressive symptoms. This study found that the volume of leisure-time exercise in sedentary workers is associated with the risk of depressive symptoms, although the relationship between exercise volume and depressive symptoms may plateau, with no additional benefits observed as the volume of exercise increases. Compared to individual exercisers, group exercisers have a lower risk of depressive symptoms. After controlling for potential confounding factors, the benefits of group exercise over individual exercise remained significant. By elucidating the nuanced relationship between exercise volume, social context, and mental health outcomes, this study has practical implications for workplace health policies. Employers and policymakers can leverage these findings to develop structured exercise programs that prioritize group-based activities, fostering social cohesion while enhancing mental well-being.

Among group exercisers, this study found a higher proportion of individuals with high exercise volume, as well as males, younger individuals, and those with a normal weight. The higher proportion of frequent exercisers in the group exercise category may be attributed to the environment and atmosphere of group exercise, which enhance both the intensity and duration of individual participation, thereby promoting higher levels of physical activity. The higher proportion of men may be attributed to societal gender role distinctions. Men are often encouraged to engage in competitive and collective exercises, such as basketball. Men often experience a stronger sense of identity and influence within the group, receiving positive social feedback (37), which may explain why some men are more likely to participate in group exercise. A relatively large proportion of young people participate in group exercise, which may be attributed to their physiological and physical advantages. Young people are better able to withstand high-intensity activities in group exercise (e.g., fast running, intense confrontations) and recover effectively. Individuals who are not overweight or obese may have greater physical strength and overall health, which enables them to adapt more effectively during group exercise.

In the final regression model (Model 3) of this study, women exhibited a higher risk of depressive symptoms compared to men, with an OR of 2.133 (95% CI: 1.349, 3.373), consistent with findings from similar studies (38). Female professionals often bear the pressures associated with multiple roles, including family, work, and social relationships. They may be more susceptible to experiencing “role conflict,” which refers to the difficulty in balancing career and family responsibilities, thereby increasing the risk of depressive symptoms (39). In this study, individuals with higher levels of education demonstrated a lower risk of depressive symptoms. The OR for high school graduates was 0.496 (95% CI: 0.283, 0.867), and for individuals with a college education or higher, it was 0.447 (95% CI: 0.262, 0.760), suggesting that higher education serves as a “protective factor” against depressive symptoms. Other surveys have reported similar findings, indicating a negative correlation between education level and depressive symptoms. A survey of psychiatric nurses indicated that, compared to those with higher education, nurses with lower educational levels exhibited more severe depressive symptoms (40). Education may enhance professionals’ cognitive abilities, facilitating a more objective understanding of depression. Individuals with greater educational experience are likely to possess a deeper knowledge base and scientific understanding, which may aid them in accurately confronting their situation and occupational pressures. Moreover, a higher educational experience may encourage professionals to make more strategic use of personal and social resources to mitigate stressors contributing to depressive symptoms, thereby protecting their mental health (41). This study found that smokers had a higher risk of depressive symptoms compared to non-smokers, with an OR of 2.680 (95% CI: 1.755, 4.092). Other studies have also confirmed the high incidence of depressive symptoms among smokers (42). Research has demonstrated that smokers are at a higher risk of mental health issues, possibly due to similar biological and psychological mechanisms underlying both smoking and depression. Additionally, smokers often engage in other unhealthy behaviors that negatively impact their depressive symptoms (43).

This study partially supports the hypothesis that the risk of depressive symptoms in sedentary workers decreases with increased leisure-time exercise volume. A statistically significant difference was observed in the detection rate of depressive symptoms across individuals with varying volume of leisure-time exercise. The detection rate of depressive symptoms was higher in the low-exercise group compared to the medium and high exercise groups. However, no statistically significant difference was found between the medium and high exercise groups. Logistic regression analysis revealed that the OR for the medium exercise group was 0.517 (95% CI: 0.351, 0.761), and for the high exercise group, it was 0.559 (95% CI: 0.354, 0.880). The OR did not decrease as exercise volume increased, suggesting that the mental health benefits of leisure-time exercise may not improve with further increases in exercise volume.

Based on these findings, we speculate that the notion that more exercise is always beneficial for mental health may not hold beyond a certain threshold. Previous studies have indicated a roughly “U”-shaped relationship between exercise frequency and mental health burden (24), suggesting that a higher volume of exercise is not always linked to improved mental health outcomes. The volume of exercise does not necessarily correlate with greater benefits, and selecting the appropriate level of exercise is crucial for promoting mental health.

The hypothesis that sedentary workers who participated in group exercise had a lower risk of depressive symptoms compared with individual exercise, was supported by this study. The detection rate of depressive symptoms was lower in group exercisers than in individual exercisers, with the difference being statistically significant (*P* < 0.05). Logistic regression analysis demonstrated that, after controlling for sociodemographic characteristics and exercise volume, the benefit of group exercise in reducing depressive symptoms compared to individual exercise persisted, with an OR of 0.624 (95% CI: 0.420, 0.927). Research has shown that group exercisers tend to have better mental health than individual exercisers (25). Additionally, the benefits of team sports as the primary exercise method extends across the lifespan, from adolescence (44) to old age (45). Engaging in group exercise during leisure time may positively impact depressive symptoms, potentially due to the social and mental health benefits associated with group participation. Research indicates that individuals who participate in sports emphasizing collaboration, friendship, and teamwork report higher levels of collective belonging and social connection. Such participants have the opportunity to build peer support within the social environment of group exercise, thus enhancing their mental health (46). As group exercise fosters a social network, group exercisers can offer companionship and support to some extent. Team exercise may also require communication skills, which can enhance social interaction and confidence, a factor in reducing depressive symptoms (47). When utilizing exercise as an intervention for depression in sedentary workers, it is important to recognize the advantages of group exercise, while also acknowledging the benefits of individual exercise. There is no universally suitable exercise for all participants. The choice of exercise method may depend on an individual’s physical condition, preferences, and the availability of community infrastructure. Selecting an exercise modality that is enjoyable and sustainable over time is beneficial to the mental health of sedentary workers.

### 4.1 Strengths and limitations

This study stands out from previous research in several ways. (1) By focusing on sedentary workers—a population particularly vulnerable to depression due to occupational constraints—our study moves beyond general exercise-mental health research to a more context-specific investigation. (2) Our findings challenge the assumption that “more exercise is always better” by demonstrating that the mental health benefits of exercise may plateau beyond a certain volume. This insight refines current exercise recommendations by emphasizing the importance of moderate, sustainable activity rather than excessive exercise. (3) We provide empirical evidence that group exercise is more effective than individual exercise in reducing depressive symptoms, likely due to its social support and engagement benefits. This distinction is crucial for designing workplace wellness programs that encourage social interaction alongside physical activity. Limitations of the study include: (1) Given the cross-sectional nature of this study, causality cannot be inferred. (2) The survey relies on self-reported data, which may be subject to recall and social desirability biases. (3) The study only distinguished between individual and group exercise, without examining the effects of specific exercise programs on depressive symptoms in greater detail. (4) As the study was conducted among sedentary workers from a single region in China, the findings may not be generalizable to other cultural or occupational contexts. (5) The study does not consider factors such as diet, sleep, work stress and social support, which may influence depressive symptoms.

Future research directions include: (1) Examining the relationship between occupational characteristics of sedentary workers, additional influencing factors such as diet, sleep, work stress and social support for depressive symptoms, to provide a comprehensive foundation for developing more effective mental health intervention strategies. (2) Further research could investigate the potential mechanisms underlying the role of group exercise in reducing depression risk, such as the influence of social networks and other factors, to better inform practice. (3) Randomized controlled trials could be employed to assess the effectiveness of individual and group exercise participation in alleviating depressive symptoms among sedentary workers. Longitudinal or experimental designs should be carried out to establish a clearer causal relationship between leisure-time exercise and depressive symptoms in sedentary workers. (4) Objective measures such as accelerometers and clinical assessments should be incorporated to improve data accuracy. (5) Future studies should include participants from diverse cultural and occupational backgrounds to improve external validity.

## 5 Conclusions

Previous studies have shown that exercise is associated with a reduced risk of depressive symptoms. The results of this study suggest that the benefits of exercise on depressive symptoms in sedentary workers do not always increase with higher volumes of exercise. Moreover, compared to individual exercisers, group exercisers have a lower risk of depressive symptoms. This study significantly advances understanding of how leisure-time exercise mitigates depressive symptoms in sedentary workers and paves the way for more effective, evidence-based mental health strategies tailored to sedentary professionals.

## Data Availability

The raw data supporting the conclusions of this article will be made available by the authors, without undue reservation.
